# Development of a Phage-Displayed Nanobody-Based Competitive Immunoassay for the Sensitive Detection of Soybean Agglutinin

**DOI:** 10.3390/foods13121893

**Published:** 2024-06-16

**Authors:** Menghan Zhang, Yulou Qiu, Ajuan You, Siyi Song, Qin Yang, Biao Zhang, Xianshu Fu, Zihong Ye, Xiaoping Yu

**Affiliations:** Zhejiang Provincial Key Laboratory of Biometrology and Inspection & Quarantine, College of Life Science, China Jiliang University, Hangzhou 310018, China; mhzhang0920@163.com (M.Z.); youajuan@163.com (A.Y.); 17816122046@163.com (S.S.); 13540527927@163.com (Q.Y.); zhangbiao9129@163.com (B.Z.); fxs@cjlu.edu.cn (X.F.); zhye@cjlu.edu.cn (Z.Y.); yxp@cjlu.edu.cn (X.Y.)

**Keywords:** soybean agglutinin, phage-displayed nanobody, competitive immunoassay, streptavidin poly-HRP

## Abstract

Soybean agglutinin (SBA) is a primary antinutritional factor in soybeans that can inhibit the growth of humans and mammals, disrupt the intestinal environment, and cause pathological changes. Therefore, detecting and monitoring SBA in foods is essential for safeguarding human health. In this paper, M13 phage-displayed nanobodies against SBA were isolated from a naive nanobody library. An M13 phage-displayed nanobody-based competitive enzyme-linked immunosorbent assay (P-cELISA) was then established for SBA analysis using biotinylated anti-M13 phage antibody (biotin-anti-M13) and streptavidin poly-HRP conjugate (SA-poly-HRP). The biotin-anti-M13@SA-poly-HRP probe can easily amplify the detection signal without the chemical modifications of phage-displayed nanobodies. The established P-cELISA presented a linear detection range of 0.56–250.23 ng/mL and a limit of detection (LOD) of 0.20 ng/mL, which was 12.6-fold more sensitive than the traditional phage-ELISA. Moreover, the developed method showed good specificity for SBA and acceptable recoveries (78.21–121.11%) in spiked wheat flour, albumen powder, and whole milk powder. This study proposes that P-cELISA based on biotin-anti-M13@SA-poly-HRP may provide a convenient and effective strategy for the sensitive detection of SBA.

## 1. Introduction

Soybean is one of the most important agro-products worldwide. It contains high-quality protein, oil, and other nutrients and is used as a main source of food for humans and livestock [1,2,3]. However, the antinutritional factors (such as soybean globulin, trypsin inhibitor, and lectin) in soybeans hinder the digestion of nutrients and may pose potential threats to human and livestock health [4,5,6,7]. SBA is a main antinutritional factor in soybeans and their byproducts. This tetrameric glycoprotein with a molecular weight of approximately 120 kDa is resistant to digestive enzymes and dry heat treatment [8,9]. SBA exhibits its antinutritional effects by interacting with the receptor proteins on the membrane of intestinal epithelial cells. The negative effects can cause growth inhibition, intestinal damage, and pathological changes [10,11,12]. Hence, establishing sensitive and accurate approaches for detecting and monitoring SBA in foods is crucial.

Several methods have been applied for SBA detection. The classical approach is the erythrocyte agglutination test, which is mainly used for qualitative or semiquantitative detection and has the drawbacks of a complicated operation and poor repeatability. Quantitative methods have also been developed for SBA detection, including liquid chromatography–mass spectrometry [13], electrochemical biosensors [14,15,16,17], and ELISAs [18,19,20,21]. Among these, ELISAs are simple, specific, and low cost, making them a useful tool for detecting soybean proteins in foods [22,23]. Morishita et al. [24] developed a sandwich immunoassay based on polyclonal antibodies (pAbs) for the determination of soybean proteins in processed foods and achieved an LOD of 0.47 ng/mL. Li [25] developed an indirect ELISA for detecting soybean lectin using mouse monoclonal antibodies (mAbs), showing an LOD of 30 ng/mL. These ELISAs were developed based on traditional mAbs and pAbs. Nevertheless, the preparation of traditional antibodies is complex and time consuming, and it has a poor batch-to-batch reproducibility [26,27,28].

For decades, nanobodies have been widely used as alternatives to traditional antibodies in immunoassays [29,30,31]. These single-domain antibody fragments from the heavy-chain-only antibody were originally discovered in camelid species. They are the smallest antigen-binding antibody, with a size of approximately 15 kDa [32,33,34]. Compared with traditional antibodies, nanobodies provide the properties of high affinity and specificity, can recognize inaccessible epitopes, and can be effectively selected by phage display technology in 1–2 weeks [35]. In addition, M13 phage-displayed nanobodies have both nanobody characteristics and M13 phage features, which have been applied in immunoassays. M13 phage is a filamentous bacteriophage, with a size of 880 × 6 nm [36]. It consists of 3–5 copies of minor proteins (pIII, pVI, pVII, and pIX) and 2700 copies of pVIII major capsid proteins [37]. The pIII proteins at one end of the phage are responsible for molecular recognition, whereas the thousands of pVIII capsid proteins on the surface can react with probes for signal amplification [38]. Therefore, M13 phage-displayed nanobodies could be a favorable immunoreagent for the sensitive detection of target analytes.

Generally, the amplification of a detection signal is an effective strategy to improve the sensitivity of immunoassays. The detection signal can be amplified using the biotin–streptavidin system, poly-enzyme conjugate, and novel nanoparticle probes [39,40,41]. Due to the high affinity between biotin and streptavidin, the biotin–streptavidin system has been widely used in immunoassays for sensitivity improvement. Sun et al. [39] developed a biotinylated nanobody and streptavidin–HRP-based ELISA for the detection of ochratoxin A in cereal, and the sensitivity of the method was improved 10 times compared with the traditional ELISA. Lu et al. [40] developed a plasmonic colorimetric immunosensor for zearalenone detection using poly-HRP amplification, exhibiting an LOD of 0.04 ng/mL. The introduction of poly-HRP increased the amount of enzyme and generated more catalytic capacity.

**Figure 1:**
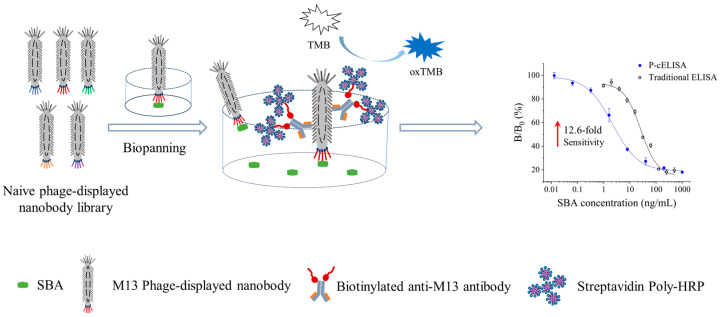
Schematic of the phage-displayed nanobody-based P-cELISA.

Herein, M13 phage-displayed nanobodies specifically binding to SBA were screened from a naive phage-displayed nanobody library. A biotin-anti-M13@SA-poly-HRP-based P-cELISA was established for SBA detection using the specific phage-displayed nanobody as the recognition element and combining biotin-anti-M13 with SA-poly-HRP as the signal reporter (Figure 1). Owing to its special structure, the M13 phage surface provided multiple binding epitopes for biotin-anti-M13. The SA-poly-HRP was then closely bound to biotin-anti-M13 and carried a high amount of HRP on the M13 phage to enhance the detection signal. The biotin-anti-M13@SA-poly-HRP-based phage-ELISA effectively improved the sensitivity of traditional phage-ELISA without chemical modifications, exhibiting a favorable LOD and linear range for SBA. The established method was further evaluated by estimating its specificity and applying it for detection in real food samples, indicating its potential in practical applications. The proposed P-cELISA may offer an alternative strategy for the determination of SBA and may be applied to the analysis of other hazards.

## 2. Materials and Methods

### 2.1. Materials and Reagents

The naive nanobody library, *Escherichia coli* TG1, helper phage M13KO7, biotinylated anti-M13 antibody, and HRP-conjugated anti-M13 antibody were obtained from AlpVHHs Co., Ltd. (Chengdu, China). Soybean agglutinin (purity ≥ 90%) was purchased from Sigma-Aldrich (St. Louis, MO, USA). The 96-well microplates and skim milk powder were purchased from Sangon Biotech (Shanghai, China). The streptavidin poly-HRP conjugate was purchased from MyBioSource Co., Ltd. (San Diego, CA, USA). The TMB substrate and stop solution for the TMB substrate were purchased from Beyotime (Shanghai, China). The soybean agglutinin ELISA kit was purchased from Nanjing enzyme-linked Biotechnology Co., Ltd. (Nanjing, China). Food samples (wheat flour, protein powder, and whole milk powder) were purchased from a local market (Hangzhou, China). Unless otherwise specified, the remaining chemicals used in this study were of analytical grade. Ultrapure water was obtained using the Milli-Q system (Billerica, MA, USA).

### 2.2. Biopanning of Phage-Displayed Nanobodies

The biopanning of anti-SBA phage-displayed nanobodies was carried out according to previously reported methods [42,43]. First, antigen SBA was diluted with 0.01 M PBS to 100 μg/mL and placed onto a microtiter plate (100 μL/well) for incubation at 4 °C overnight. The SBA-coated microplates were washed three times using 0.01 M PBS with 0.1% Tween-20 (0.1% PBST) after which 3% BSA-PBS was added at 37 °C for 2 h to block the excess sites. Thereafter, the microplates were incubated with naive phage-displayed nanobody library (2 × 10^11^ pfu) at 37 °C for 1 h. Nonspecific-binding phages were washed away using 0.01 M PBS and 0.1% PBST alternately five times. Afterward, 100 μL/well of 0.2 M Gly-HCl (containing 0.1% BSA, pH 2.5) was added to the microplates to elute the bound phages at 37 °C for 8 min under gentle shaking. The elution was instantly added to 15 μL of neutralization buffer (1 M Tris-HCl, pH 9.0), and the eluted phages were used for titer measurement and amplification for the next screening. For the subsequent rounds of screening, the concentration of antigen SBA was decreased to 50, 25, and 12.5 μg/mL. The SBA-coated wells were added to the amplified phage-displayed nanobody libraries and kept at 2 × 10^11^ pfu. The washing times were gradually increased to 10, 15, and 20 times.

### 2.3. Identification of Phage-Displayed Nanobodies

After biopanning, 48 individual phages from the last round of titration plates were picked up and determined by phage-ELISA. First, the 48 randomly selected clones were inoculated in 1 mL 2 × YT culture medium containing 100 μg/mL ampicillin (Amp) and grown overnight at 220 rpm and 37 °C. After that, the cells were inoculated in 1 mL 2 × YT culture medium supplemented with 2% glucose and 100 μg/mL Amp with a 1% inoculation dosage, and they were incubated at 220 rpm at 37 °C to reach the logarithmic growth phase. Then, the M13KO7 helper phages with a cell-to-phage ratio of 1:20 were added into culture medium for rescuing for 45 min at 220 rpm and 37 °C. After centrifugation at 1000× *g* for 10 min at 4 °C, the precipitates were re-suspended in 1 mL fresh 2 × YT medium containing 100 μg/mL Amp and 40 μg/mL kanamycin (Kana) followed by growing overnight (220 rpm, 30 °C). Thereafter, the cells were centrifuged at 8000× *g* for 10 min at 4 °C and the supernatants were used for phage-ELISA.

In brief, SBA (diluted 2 μg/mL in 0.01 M PBS, 100 μL/well) was coated into microtiter plates overnight at 4 °C. After being washed three times with PBST, 5% skim milk powder in PBS was dispensed into the plates to block the unoccupied sites for 2 h. The nanobody-displaying phages were then added for 1 h of incubation at 37 °C. After being washed three times, HRP-conjugated anti-M13 antibody (diluted 1:30,000) was added and incubated into the microtiter plates for 1 h. The plates were washed again, and the TMB substrate was transferred onto the plates for a 15 min reaction. After the reaction was stopped with stop solution (50 μL/well), OD450 was detected using a microplate reader (Allsheng, Hangzhou, China). Positive phage clones were defined as having an absorbance that was twofold that of the negative control. Finally, the nucleotide sequences of the positive phages were sequenced, and the sequencing primer was gback 5′-GCCCCCTTATTAGCGTTTGCCATC-3′.

### 2.4. Preparation of Phage-Displayed Nanobodies

The positive clones were grown in 2 × YT culture medium with 2% glucose on a rotatory shaker at 250 rpm until the OD600 was approximately 0.5. The M13KO7 helper phages were then added at a cell-to-phage ratio of 1:20 and cultured at 37 °C for 45 min with shaking. After centrifugation at 1000× *g* for 10 min at 25 °C, *E. coli* cells were resuspended in the fresh 2 × YT culture medium with double antibiotics (Amp and Kana) and incubated at 250 rpm at 30 °C overnight. After another centrifugation (8000× *g*, 10 min, 4 °C), the phage supernatants were separated and added to 1/6 volume of 20% PEG/NaCl for precipitation. The nanobody-displaying phages were then harvested via centrifugation at 10,000× *g* for 12 min at 4 °C, resuspended in 0.01 M PBS, added to an equal volume of 100% glycerin, and stored at −20 °C for use.

### 2.5. Development of P-cELISA

The prepared anti-SBA phage-displayed nanobody was utilized to establish a competitive phage-ELISA for SBA detection. The P-cELISA procedures were implemented as follows. First, the coating antigen SBA was transferred onto the microtiter plates and incubated overnight at 4 °C. After three washes with PBST, the SBA-coated wells were blocked with 5% skim milk powder in PBS for 2 h. Subsequently, 50 μL of anti-SBA phage-displayed nanobody and an equal volume of SBA (0–1000 ng/mL) were added to the wells and gently mixed for 1 h incubation. After five washes, 100 μL of biotinylated anti-M13 antibody (100 μL/well, 1:30,000 diluted in blocking buffer) was transferred to the microplates for incubation at 37 °C for 1 h. The microplates were washed six times using PBST, and SA-poly-HRP conjugate (100 μL/well, 1:8000 diluted in blocking buffer) was then added onto the plates for 30 min of incubation. After further washing, a TMB substrate solution was added to the plates at 37 °C for 15 min. Finally, 50 μL/well of stop solution for the TMB substrate was added to stop the enzyme reaction, and the OD450 was detected using a microplate reader (Allsheng, Hangzhou, China). For the optimization of the P-cELISA performance, different concentrations of coating antigen SBA (500, 250, 125, and 62.5 ng/mL) and anti-SBA phage-displayed nanobodies (1:2000, 1:4000, 1:8000, and 1:16,000) were estimated in advance.

### 2.6. Sensitivity and Selectivity of P-cELISA

To evaluate the sensitivity of the phage-displayed nanobody-based P-cELISA, a standard inhibition curve was established by plotting the binding rate (B/B_0_) against the concentration of SBA, where B and B_0_ represented the OD450 values of the positive sample containing SBA and the negative sample without SBA. The LOD value was defined as the 10% inhibitory concentration (IC_10_), and the linear detection range of the method was defined as IC_20_–IC_80_.

The selectivity of the developed P-cELISA was investigated by identifying cross-reactivities with other bean proteins, including Phaseolus vulgaris agglutinin (PHA), concanavalin (ConA), soybean protease inhibitor (STI), and urease (URE). 

### 2.7. Method Validation

Recovery tests were carried out to estimate the practical application of the developed method. SBA-negative food samples (wheat flour, whole milk powder, and protein powder) collected from local markets (Hangzhou, China) were spiked with SBA standard solution at various concentration levels (500, 1000, 2000, and 4000 μg/kg). Sample extraction and dilution were conducted following a previous method [18]. In brief, 1 g of food sample was added into 4 mL of extraction buffer (0.01 M PBS) and extracted overnight by gently shaking at 4 °C. After centrifugation at 9000× *g* for 15 min, the extraction supernatants were collected and then diluted five times with 0.01 M PBS for P-cELISA analysis. For the validation of the developed P-cELISA, the samples were also analyzed using an SBA commercial ELISA kit, and the results between the two methods were evaluated.

## 3. Results and Discussion

### 3.1. Panning and Identification of SBA-Specific Nanobodies 

**Figure 2:**
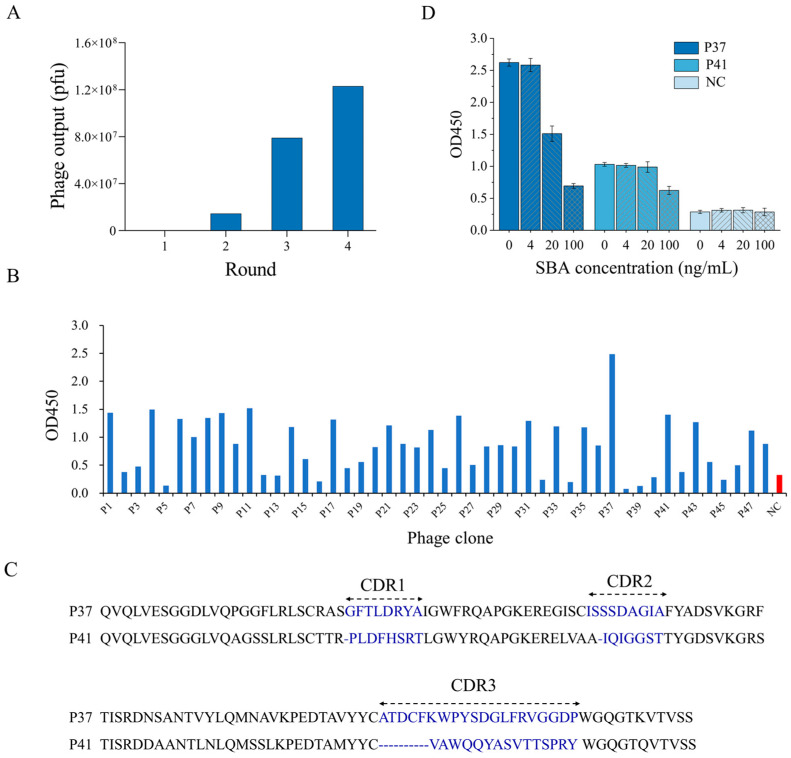
(**A**) Number of phage outputs in each round of panning. (**B**) Identification of anti-SBA phage clones using phage-ELISA. NC, negative control. (**C**) Amino acid sequences of the positive clones. (**D**) Identification of the positive phage clones using competitive phage-ELISA. NC, negative control.

After panning, the number of output phages increased from 3.3 × 10^5^ pfu in the first round to 1.2 × 10^8^ pfu in the fourth round, revealing that the output phages against SBA were effectively enriched 364-fold (Figure 2A). Meanwhile, 48 individual phage clones were randomly picked up from the titration plates of the last round of biopanning and identified via indirect phage-ELISA. As presented in Figure 2B, 28 of the phage-displayed nanobody clones were determined to specifically bind to SBA, and their OD450 was twofold higher than that of the negative control. The gene sequencing results indicated that two phage clones (P37 and P41) were obtained. Figure 2C illustrates that the amino acids in the framework region of P37 and P41 were highly homologous, and those in the complementarity-determining region (CDR) were different. In addition, the CDR3 of P37 was longer than that of P41. The diversity in the CDR regions of the nanobodies may have resulted in differences in their binding activity. As shown in Figure 2D, phage clones P37 and P41 can bind to the coated SBA and compete with free SBA. Among them, P37 exhibited a higher sensitivity and was selected for further research. 

### 3.2. Development of P-cELISA 

**Figure 3:**
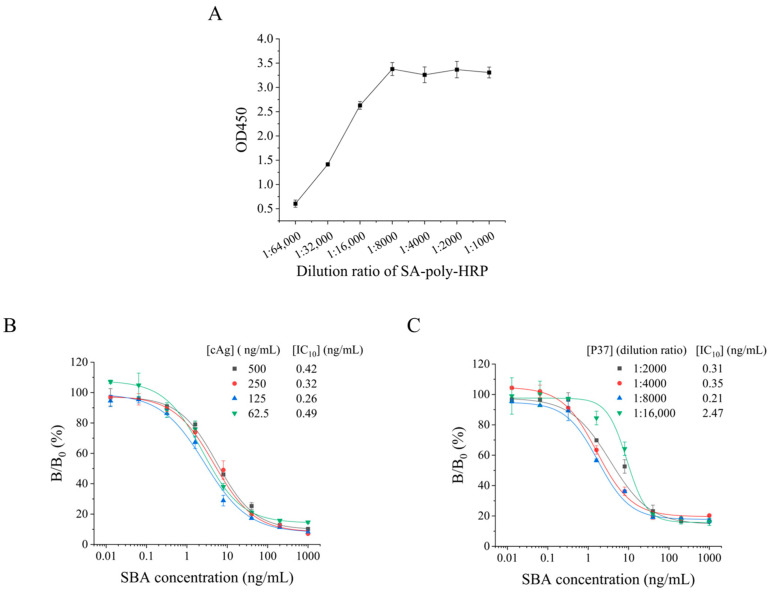
Optimization of conditions for P-cELISA. (**A**) SA-poly-HRP saturation concentration, (**B**) coating antigen concentration (cAg), and (**C**) dilution ratio of the phage-displayed nanobody.

A phage-displayed nanobody-based competitive ELISA was developed for SBA detection. Biotin-anti-M13 and SA-poly-HRP were used as signal reporters to improve the sensitivity of the P-cELISA. First, the saturation concentration of SA-poly-HRP was evaluated to maximize the use of SA-poly-HRP for signal amplification. Figure 3A shows that the absorbance value gradually increased when the dilution ratio of the SA-poly-HRP ranged from 1:64,000 to 1:8000 and remained almost the same when the dilution ratio ranged from 1:8000 to 1:1000. Therefore, a dilution ratio of 1:8000 was selected as the saturation concentration of the SA-poly-HRP. For further optimization, different concentrations of coating antigen SBA (500, 250, 125, and 62.5 ng/mL) and phage-displayed nanobody P37 (1:2000, 1:4000, 1:8000, and 1:16,000) were estimated. As shown in Figure 3B, properly reducing the concentrations of the coating antigen can improve the sensitivity. When the coating SBA concentration was 125 ng/mL, the IC_10_ was the lowest. Therefore, the optimum concentration of coating antigen was 125 ng/mL. Figure 3C shows that the lowest IC_10_ was obtained at a dilution ratio of 1:8000. Therefore, 1:8000 was determined as the optimum concentration of phage-displayed nanobody P37.

**Figure 4:**
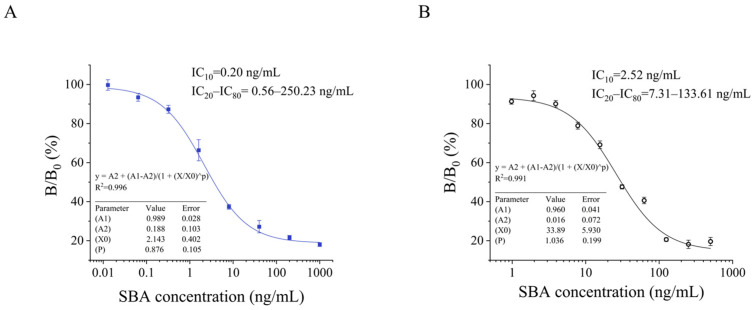
Standard inhibition curve of (**A**) P-cELISA and (**B**) traditional phage-ELISA for SBA detection.

Under the optimized conditions, the biotin-anti-M13@SA-poly-HRP-based P-cELISA exhibited a linear detection range of 0.56–250.23 ng/mL and an IC_50_ of 3.51 ng/mL. The LOD calculated as IC_10_ was 0.20 ng/mL (Figure 4A). For comparison, the traditional HRP-labeled anti-M13 antibody-based phage-ELISA was developed with a linear range of 7.31–133.61 ng/mL and an LOD of 2.52 ng/mL (Figure 4B). The results showed that the sensitivity of the proposed biotin-anti-M13@SA-poly-HRP-based P-cELISA exhibited a 12.6-fold improvement over that of the traditional phage-ELISA.

Phage-displayed nanobodies integrate the advantages of nanobodies and M13 phage, making them an excellent material in immunoassays. They also have the characteristics of a high affinity, easy preparation, high reproducibility, and low cost. Moreover, the 2700 copies of capsid pVIII protein on the surface of the M13 phage provide plenty of epitopes to probes for signal enhancement. With the phage-displayed nanobody as the detection antibody and biotin-anti-M13@SA-poly-HRP as the tracer, the established P-cELISA provides an effective strategy for the sensitive detection of SBA. No additional chemical modifications of the phage-displayed nanobody are required, and the biotin-anti-M13@SA-poly-HRP-based P-cELISA could be easily applied for the detection of other proteins and small molecules.

### 3.3. Cross-Reactivity 

**Figure 5:**
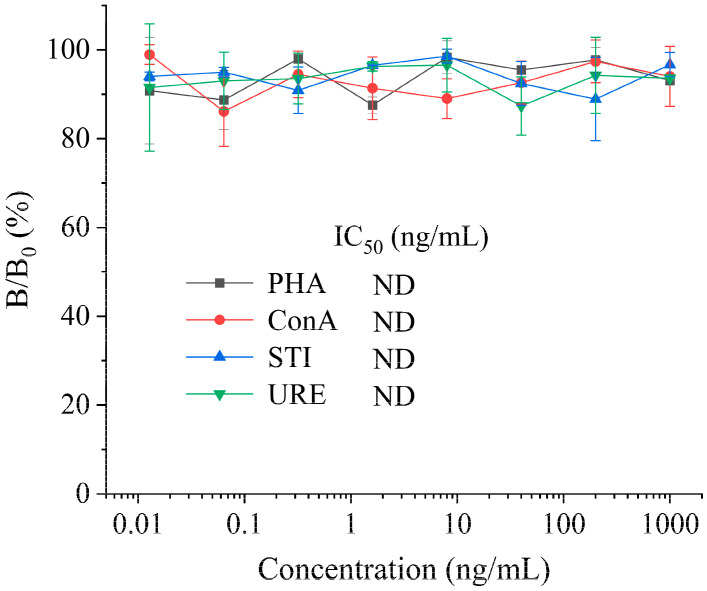
Cross-reactivity of P-cELISA with other bean proteins.

The selectivity of P-cELISA was investigated by detecting other bean proteins, including PHA, ConA, STI, and URE. The cross-reactivities were determined using the formula CR% = (IC_50_ of SBA)/(IC_50_ of other bean proteins) × 100%. The results are shown in Figure 5. Under the optimized conditions, negligible cross-reactivities were measured with other bean proteins, indicating that the established P-cELISA has a good specificity for the detection of SBA.

### 3.4. Sample Analysis and Validation 

**Figure 6:**
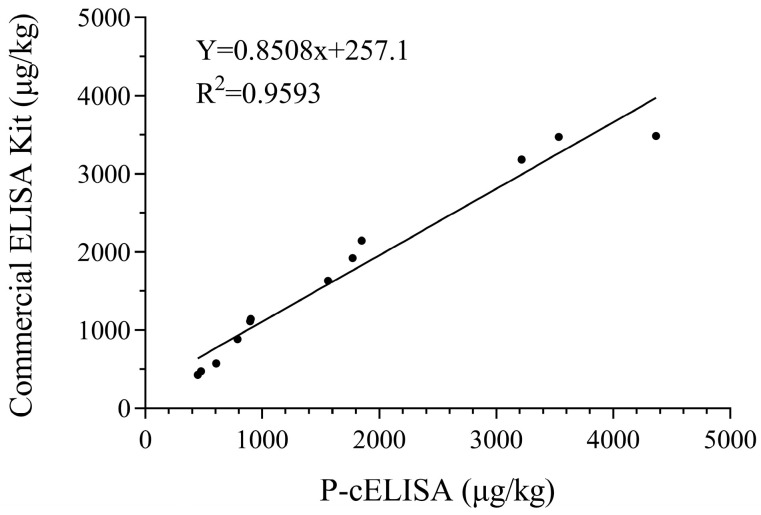
Correlation of results obtained by P-cELISA and commercial ELISA kit.

Matrix interference is a common challenge in immunoassays for food sample analysis. It can interfere with the binding between the antibody and antigen, thereby affecting the practicability of immunoassays. The dilution of sample extracts is a common way to reduce food matrix interference. The matrix interference of three possible soybean-supplemented food samples (wheat flour, albumen powder, and whole milk powder) was observed. Negligible interference was found with a 20-fold dilution of extracts. A spike and recovery analysis was performed to evaluate the accuracy and precision of the established P-cELISA. The SBA-free wheat flour, albumen powder, and whole milk powder spiked with known concentrations (500, 1000, 2000, and 4000 μg/kg) of SBA standard solution were assessed using P-cELISA, and the results are shown in Table 1. The average recoveries of SBA in the wheat flour ranged from 88.33% to 121.11%, with a coefficient of variation (CV) in the range of 1.95–8.66%. The recoveries in the albumen powder ranged from 78.98% to 92.32%, and the CV was in the range of 1.47–10.84%. In addition, the recoveries in the whole milk powder ranged from 78.21% to 109.17%, with a CV of 1.61–4.22%. The food samples were also analyzed using an SBA commercial ELISA kit for validation. As presented in Figure 6, the results from the P-cELISA and commercial ELISA kit were in agreement with each other, showing a correlation coefficient of 0.9593. 

## 4. Conclusions

Phage-displayed nanobodies specifically binding to SBA were first isolated from a naive phage-displayed nanobody library. A phage-displayed nanobody-based competitive immunoassay using biotin-anti-M13@SA-poly-HRP was then developed for the determination of SBA. The working range of the developed P-cELISA was 0.56–250.23 ng/mL. P-cELISA achieved an LOD of 0.20 ng/mL, showing a 12.6-fold higher sensitivity than the conventional phage-ELISA. Furthermore, the developed method exhibited good accuracy and precision for SBA detection in food samples. Phage-displayed nanobodies are a promising immunoreagent that can be continuously produced on a large scale, offering advantages in terms of rapid production, low cost, and high purity. In addition, thousands of capsid pVIII proteins on the phage surface can easily combine with biotin-anti-M13@SA-poly-HRP probes to amplify the detection signal without any chemical modifications. The results demonstrated that the proposed P-cELISA is a reliable and practical method for sensitive SBA detection and may provide a potential universal strategy for detecting other analytes.

## Figures and Tables

**Table 1:** Recovery of SBA spiked in food samples detected by P-cELISA (*n* = 3).

Samples	Added (μg/kg)	This Work			Commercial ELISA Kit		
		Detected (μg/kg)	Recovery (%)	CV (%)	Detected (μg/kg)	Recovery (%)	CV (%)
Wheat flour	500	605.55 ± 23.23	121.11	3.84	428.04 ± 9.18	85.61	2.14
	1000	902.13 ± 78.12	90.21	8.66	1140.31 ± 20.73	114.03	1.82
	2000	1770.62 ± 34.54	88.53	1.95	2144.50 ± 97.13	107.23	4.53
	4000	3533.20 ± 117.78	88.33	3.33	3182.52 ± 92.99	79.56	2.92
Albumen powder	500	447.83 ± 48.56	89.56	10.84	475.92 ± 68.43	95.67	14.3
	1000	789.82 ± 9.51	78.98	1.47	884.01 ± 62.24	88.56	7.04
	2000	1846.48 ± 189.78	92.32	10.27	1923.47 ± 45.21	96.23	2.35
	4000	3214.88 ± 78.98	80.32	2.46	3482.66 ± 173.15	87.11	4.97
Whole milk powder	500	475.86 ± 7.67	95.17	1.61	574.09 ± 57.58	115.12	10.02
	1000	897.80 ± 37.89	89.78	4.22	1114.48 ± 49.30	111.78	4.43
	2000	1564.20 ± 56.78	78.21	3.63	1631.10 ± 41.36	82.33	2.54
	4000	4366.84 ± 178.64	109.17	4.08	3472.65 ± 58.20	87.23	1.68

## Data Availability

The original contributions presented in the study are included in the article, further inquiries can be directed to the corresponding author.
